# Comparative Genomics of *Bacillus thuringiensis* Reveals a Path to Specialized Exploitation of Multiple Invertebrate Hosts

**DOI:** 10.1128/mBio.00822-17

**Published:** 2017-08-08

**Authors:** Jinshui Zheng, Qiuling Gao, Linlin Liu, Hualin Liu, Yueying Wang, Donghai Peng, Lifang Ruan, Ben Raymond, Ming Sun

**Affiliations:** a State Key Laboratory of Agricultural Microbiology, Huazhong Agricultural University, Wuhan, China; b College of Informatics, Huazhong Agricultural University, Wuhan, China; c College of Life and Environmental Science, University of Exeter, Penryn, United Kingdom; The Sanger Institute

**Keywords:** *Bacillus thuringiensis*, host specialization, invertebrate pathogen, population genomics

## Abstract

Understanding the genetic basis of host shifts is a key genomic question for pathogen and parasite biology. The *Bacillus cereus* group, which encompasses *Bacillus thuringiensis* and *Bacillus anthracis*, contains pathogens that can infect insects, nematodes, and vertebrates. Since the target range of the essential virulence factors (Cry toxins) and many isolates is well known, this group presents a powerful system for investigating how pathogens can diversify and adapt to phylogenetically distant hosts. Specialization to exploit insects occurs at the level of the major clade and is associated with substantial changes in the core genome, and host switching between insect orders has occurred repeatedly within subclades. The transfer of plasmids with linked *cry* genes may account for much of the adaptation to particular insect orders, and network analysis implies that host specialization has produced strong associations between key toxin genes with similar targets. Analysis of the distribution of plasmid minireplicons shows that plasmids with *orf156* and *orf157*, which carry genes encoding toxins against Lepidoptera or Diptera, were contained only by *B. thuringiensis* in the specialized insect clade (clade 2), indicating that tight genome/plasmid associations have been important in adaptation to invertebrate hosts. Moreover, the accumulation of multiple virulence factors on transposable elements suggests that cotransfer of diverse virulence factors is advantageous in terms of expanding the insecticidal spectrum, overcoming insect resistance, or through gains in pathogenicity via synergistic interactions between toxins.

## IMPORTANCE

Population genomics have provided many new insights into the formation, evolution, and dynamics of bacterial pathogens of humans and other higher animals, but these pathogens usually have very narrow host ranges. As a pathogen of insects and nematodes, *Bacillus thuringiensis*, which produces toxins showing toxicity to many orders of insects and other invertebrates, can be used as a model to study the evolution of pathogens with wide host ranges. Phylogenomic analysis revealed that host specialization and switching occur at the level of the major clade and subclade, respectively. A toxin gene co-occurrence network indicates that multiple toxins with similar targets were accumulated by the same cell in the whole species. This accumulation may be one of the strategies that *B. thuringiensis* has used to fight against host resistance. This kind of formation and evolution of pathogens represents a different path used against multiple invertebrate hosts from that used against higher animals.

## INTRODUCTION

Many pathogens originate from nonpathogenic ancestors by acquiring virulence genes through horizontal gene transfer (HGT) (1). Virulence factors (VFs) have a range of functions to ensure successful infection and survival in host environments. The evolution of pathogenicity does not occur in a static environment, since hosts are capable of developing resistance or acquiring immunity to prevalent infectious agents (2). In turn, pathogens can evolve new methods to overcome host resistance (3, 4). Mutations that overcome resistance can impair pathogen infectivity in previously susceptible hosts, leading to trade-offs and specialization in particular genotypes (5, 6). In a community with a wide range of possible hosts, coevolutionary dynamics can lead to host switching or “coevolutionary alternation,” as parasites may be favored to specialize in the currently least well-defended host (7). If host switching is rapid, as predicted by coevolutionary theory, and requires relatively few allelic or genomic changes, then closely related pathogens would be expected to be able to exploit a wide taxonomic range of hosts. Conversely, if host switching by pathogens requires multiple new VFs or many allelic changes, then pathogen clades may typically exploit a relatively narrow taxonomic range of hosts.

Comparative genomics has provided many new insights into the formation, evolution, and adaptation of some bacterial pathogens and provided evidence for both of these scenarios. For *Salmonella*, most serotypes are host specialized, causing diseases in certain hosts, and strains with similar host ranges are clustered together phylogenetically, with different combinations of *Salmonella* pathogenicity islands (PAIs) and different functional losses, respectively (8, 9). In *Campylobacter*, different lineages tend to be specialized for different vertebrate taxa, although ecological conditions in the farmed environment are undermining the basis of specialization (10, 11). In *Escherichia coli*, different combinations of VFs determine different pathotypes that are specialized for different host niches and can cause enteric/diarrheal disease, urinary tract infections, and sepsis/meningitis. Population genomics has shown that *E. coli* pathotypes are not restricted to specialized phylogenetic groups (12–14).

The *Bacillus cereus* group contains both pathogenic and nonpathogenic bacteria, and the taxonomic range of hosts susceptible to this group strains includes three phyla (Arthropoda, Nematoda, Chordata) (15, 16), making this group a powerful system for investigating the genomics of host specialization and changes in host range. The group includes *Bacillus anthracis*, which specializes in grazing ungulate herbivores (17, 18); *B. cereus sensu stricto*, an opportunistic intestinal pathogen of vertebrates (19); and *Bacillus thuringiensis*, which is an invertebrate specialist infecting nematodes and three insect orders. *B. cereus* and *B. thuringiensis* are currently differentiated on the basis of the production of crystal (Cry) toxin inclusion bodies, the genes for which are located predominantly on plasmids (20). However, previous phylogenetic analysis has identified different major clades in the *B. cereus* group that are ecologically and genetically distinct. In particular, the two major pathogen clades vary in their propensity to cause serious infections in either vertebrates or invertebrates, while the third clade, composed primarily of *Bacillus weihenstephanensis* and *Bacillus mycoides*, has a predominantly saprophytic niche (16, 21–24).

Toxins produced by *B. thuringiensis* that play a major role in the targeting of insects and other invertebrates include insecticidal Cry proteins, which are obligate VFs, vegetative insecticidal protein (Vip) toxins, and cytotoxin (Cyt) proteins (25, 26). More than 750 Cry proteins from 74 families (Cry1 to Cry74) have been described; each family being composed of members with >45% amino acid identity. More than 150 Vip toxins and 38 Cyt proteins from three families have also been reported (http://btnomenclature.info/). These proteins were reported to be toxic to many orders of insects and other invertebrates, such as nematodes, mites, and protozoa. Different toxins have different targets; Cry1, Cry2, and Vip3 are highly toxic to Lepidoptera; Cry3, Cry8, and Vip1/Vip2 kill mainly Coleoptera; Cry4 and Cyt are active against Diptera insects; and Cry5, Cry6, Cry14, and Cry21 proteins are toxic to nematodes (27).

To examine the formation and evolution of host specialization in the *B. cereus* group, we built a genome-based phylogeny of *B. thuringiensis* isolates and studied the distribution and dynamics of toxin genes among these strains. We aimed to test whether adaptations to specialize on particular host taxa are phylogenetically constrained or more widely distributed and how the gain and loss of toxins have contributed to host specialization. Historically, flagellar H serotyping was a useful classification of *B. thuringiensis* isolates; and the serotypes defined by this system represent a representative sample of the diversity of this species (28). We sequenced and collected the genomes of the antiserum standard strains of all of the serotypes, including multiple strains of several serotypes. Combined with genome sequences of the *B. cereus* group obtained from GenBank, we used *B. thuringiensis* as a model to study the formation, evolution, and adaptation of bacterial pathogens.

## RESULTS

### Population structure of the *B. cereus* group.

Genome sequences from 83 serovars of *B. thuringiensis* were used in this study. Ninety-five strains were sequenced by us, including antiserum standard strains from 57 serovars (see Table S1 in the supplemental material). Genome sequences of the remaining 26 serovars were obtained from GenBank. A maximum-likelihood (ML) phylogenetic tree based on the core genome was constructed by PhyML, and population structure was determined by the Bayesian clustering method (Bayesian analysis of population structure [BAPS]). Similar methods were used to analyze the population structure of the whole *B. cereus* group on the basis of the genomes of the 140 *B. thuringiensis* and 152 other strains in this group.

10.1128/mBio.00822-17.6 TABLE S1  *B. thuringiensis* strains used in this study. Download TABLE S1, XLSX file, 0.02 MB. Copyright © 2017 Zheng et al. 2017 Zheng et al. This content is distributed under the terms of the Creative Commons Attribution 4.0 International license.

**FIG 1:**
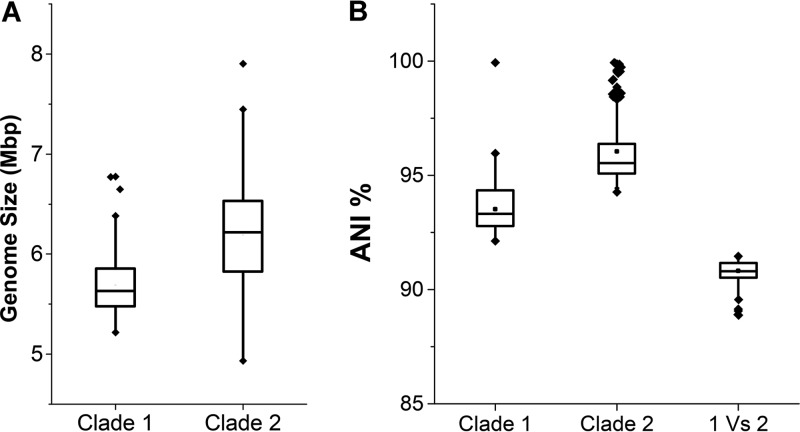
Different features of the two major clades in the *B. cereus* group. (A) Strains in clade 2 have significantly larger genomes than those in clade 1 (*P* = 3.711e-6). (B) ANIs between all pairs of strains in clade 1, between all pairs of strains in clade 2, and between strains in clades 1 and those in clade 2 (*P* < 2.2e-16).

Phylogenomic analysis divided the *B. cereus* group into four clades (Fig. S1). These clades correspond closely to groups previously described on the basis of multilocus sequence typing analysis, which uses partial sequences of seven housekeeping genes for isolate typing. Clades 1 to 3 correspond largely to the clades with those names in previous studies (16, 23, 24), with clade 1 being the “Anthracis” clade and clade 3 being the “Weihenstephanensis” clade. Most typical *B. thuringiensis* isolates were in clade 2, which can be considered as the “Thuringiensis” clade. We compared the basic genomic features of members of the two major clades (Fig. 1). Strains in clade 2 have significantly larger genomes (most of them are >6 Mbp) than those in clade 1 (usually <6 Mbp; Fig. 1A; P = 3.711 × 10^−6^ [Wilcoxon-Mann-Whitney test]), which may be due to the carriage of more plasmids in clade 2. On the phylogenetic tree, members of clade 1 had longer branches than those of clade 2, indicating greater genomic diversity within this clade. The results of average nucleotide identity (ANI) analysis support this conclusion (Fig. 1B). The ANIs of isolates in clade 2 are all >95%, significantly higher than those of isolates in clade 1 (between 92 and 95%; *P* < 2.2 ×10^−16^ [Wilcoxon-Mann-Whitney test]) and those between the members of the two clades (<92%; *P* < 2.2×10^−16^ [Wilcoxon-Mann-Whitney test]).

10.1128/mBio.00822-17.1 FIG S1  Population structure of the *B. cereus* group. Four clades are represented by four different background colors of the tree labels. *B. thuringiensis* strains are red. The binary squares and circles represent the presence and absence of different plasmids. From the innermost circle to the outermost circle, solid squares and circles represent plasmids with *orf156*/*orf157*, *rep228*, *rep466*, *repX*, *pXO1-14*/*pXO1-16*, *repA*, *ori43*, *ori44*, *ori60*, CT43_P825207-like, CT43_P851308-like, *rep165*, and pBMB2062-like minireplicons. Download FIG S1, PDF file, 1.9 MB. Copyright © 2017 Zheng et al. 2017 Zheng et al. This content is distributed under the terms of the Creative Commons Attribution 4.0 International license.

**FIG 2:**
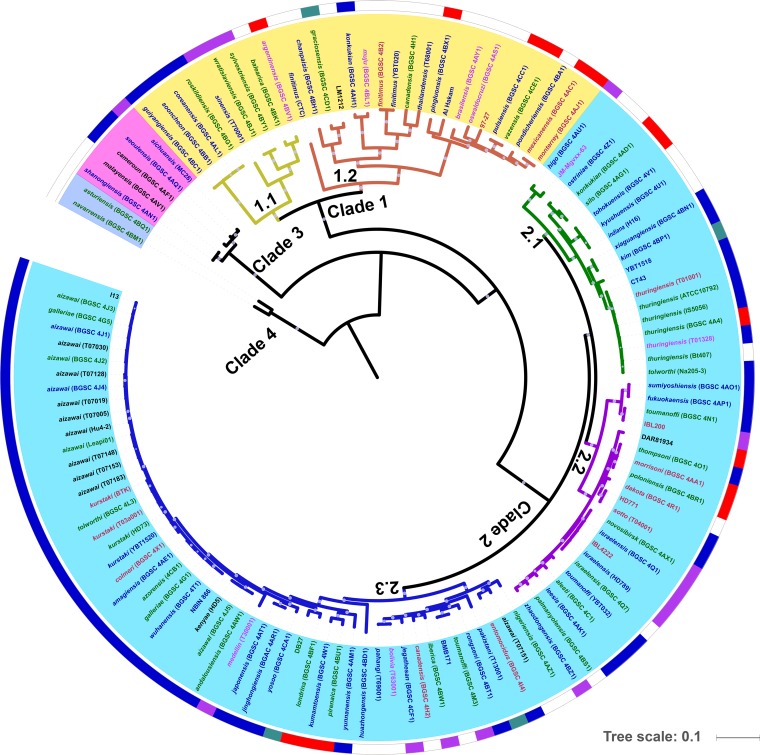
Population structure of *B. thuringiensis*. The ML tree shown was inferred by PhyML with the best model (JTT+I+G) on the basis of the alignments of the concatenated protein sequences of single-copy core genes. Bootstrap support values were calculated from 1,000 replicates, and only values of >80% are shown as white circles on the branches of the tree. The antiserum standard strains of all of the H serotypes (serovars); several strains of serotypes H1, H3abc, H5ab, and H7; and some strains with specific features were included in the analysis. The background colors of the labels represent different clades. Branches with different colors represent different lineages obtained by BAPS software. Colors represent isolated areas as follows: blue for Asia, red for Europe, green for North America, and purple for South America. The outermost circle describes the reported targets of these strains as follows: blue for Lepidoptera, purple for Diptera, dark green for nematodes, and red for Coleoptera. Target information for each strain was predicted from the host range of its toxin complement.

Though *B. thuringiensis* can be found in all clades of the *B. cereus* group, most of them (132 of 140) were clustered into two major clades (Fig. 2; Fig. S1). On the basis of BAPS analysis, clades 1 and 2 can be divided into two and three lineages, respectively (Fig. 2). We explored the diversity of *B. thuringiensis* by focusing on the phylogenetic relationships among strains with the same or different serotypes. In most cases, multiple strains of the same serovar were found in the same lineage. For example, all of the strains of serovars thuringiensis, kurstaki, aizawai, and israelensis were clustered together in lineages 2.1, 2.2, 2.3, and 2.3, respectively. In addition, many strains of different serovars clustered tightly on the basis of core gene single nucleotide polymorphism (SNP) analysis, especially in clade 2. In lineage 2.3, for example, strains of serovars kurstaki, galleriae, aizawai, tolworthi, colmeri, amagiensis, and azorensis were on the same branch, with few differences in their core gene SNPs.

### Isolates of clade 2 contain more toxin genes than those of clade 1.

**FIG 3:**
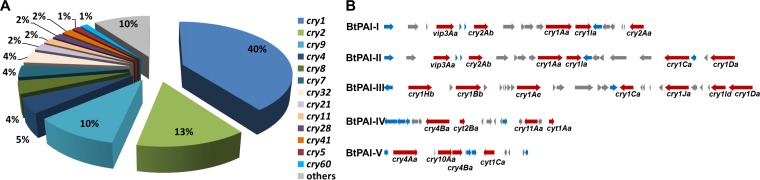
Toxin gene complements of the *B. thuringiensis* strains studied. (A) Composition of *cry* genes. (B) PAIs of the *B. thuringiensis* strains studied. Four hundred fifty-four *cry* genes from 36 rank 1 families were predicted. Red arrows represent toxin genes, and blue and gray arrows represent genes encoding transposases and genes with other functions, respectively.

As a pathogen of insects and nematodes, *B. thuringiensis* can produce three types of toxin with lethal activity, Cry, Vip, and Cyt. We focused on toxins with protein sequences >95% identical to described variants, which is the boundary representing the third rank in the nomenclature of Cry toxins. Four hundred fifty-four *cry* genes from 36 of the 74 families of rank 1 (members of each family with a protein sequence identity of >45%) reported until now were predicted (Fig. 3A). The most abundant family was *cry1*, making up 40% of the total toxin genes. Most families (23 of the 36) had <5 members of the 140 *B. thuringiensis* genomes studied here. In addition, 101 *vip* and 26 *cyt* genes were predicted (Table S2).

10.1128/mBio.00822-17.7 TABLE S2  Details about Cry proteins predicted in *B. thuringiensis*. Download TABLE S2, DOCX file, 0.02 MB. Copyright © 2017 Zheng et al. 2017 Zheng et al. This content is distributed under the terms of the Creative Commons Attribution 4.0 International license.

We analyzed the distribution of toxin genes and found that *B. thuringiensis* strains in clade 1 had significantly fewer than those in clade 2 (*P* < 2.2 × 10^−16^ [Wilcoxon-Mann-Whitney test]; Fig. S2 and Table S2). Most of the *B. thuringiensis* strains in clade 1 had only one or two *cry* genes, and no *vip* or *cyt* genes were found in these strains. Conversely, *B. thuringiensis* strains in clade 2 usually had more than two toxin genes, and many of them had more than five. Serovars containing strains with more than five toxin genes included thuringiensis and tolworthi in lineage 2.1; alesti, toumanoffi, thompsoni, and israelensis in lineage 2.2; and kurstaki, galleriae, aizawai, tolworthi, colmeri, amagiensis, and azorensis in lineage 2.3. Closely related strains could either have the same composition of toxin genes or carry divergent suites of toxin genes (Table S2). In lineage 2.1, among the six strains of thuringiensis, there were five types of toxin gene composition, with four of them containing *cry1Aa*, *cry1Ba*, *vip1Bb*, and *vip2Aa*. In israelensis of lineage 2.2, 4Q1 contained *cry60Aa* and *cry60Ba* and HD-789 had an additional seven toxin genes besides these two. In lineage 2.3, 14 strains of aizawai had seven types of toxin genes, with *cry1Da*, *cry1Ia*, *cry2Ab*, *cry9Ea*, and *vip3Aa* shared by all of these strains. Thus, the composition of toxins in *B. thuringiensis* has a very high level of diversity, implying that the gain and loss of these genes are very frequent in this species.

10.1128/mBio.00822-17.2 FIG S2  Differences between the *cry* gene numbers of the two major groups. Download FIG S2, TIF file, 0.1 MB. Copyright © 2017 Zheng et al. 2017 Zheng et al. This content is distributed under the terms of the Creative Commons Attribution 4.0 International license.

10.1128/mBio.00822-17.3 FIG S3  Co-occurrence network of all of the Cry, Cyt, and Vip proteins with known hosts from the *B. thuringiensis* Toxin Nomenclature database (http://btnomenclature.info/). Only strains with more than two toxin genes displayed in the database were analyzed. Download FIG S3, PDF file, 2.1 MB. Copyright © 2017 Zheng et al. 2017 Zheng et al. This content is distributed under the terms of the Creative Commons Attribution 4.0 International license.

### Multiple HGT events contribute to the accumulation of toxins in the same strain.

Most of the isolates in clade 2 have multiple toxin genes; and most of the genes are harbored by plasmids. We focused on the location relationships of the toxin genes among different genomes to study the dynamics of these toxins. We analyzed the putative PAIs of all of the toxin genes predicted (Fig. 3B). BtPAI-I and its variant BtPAI-II were found to be widespread in lineages 2.1 and 2.3 (Fig. 3B; Table S2). BtPAI-III, BtPAI-IV, and BtPAI-V were predicted for multiple strains of lineage 2.2 (Fig. 3B; Table S2). We suggest that many toxin genes are moved among different strains of *B. thuringiensis*, as PAIs and several toxins are usually transferred collectively.

**FIG 4:**
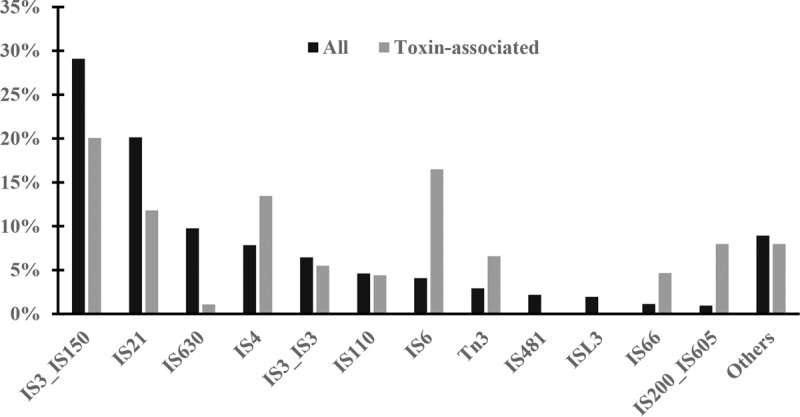
Comparison of transposase families with toxin-associated locations and other locations.

The PAIs of *B. thuringiensis* toxins commonly contain multiple transposase genes (Fig. 3B), indicating that transposons have played an important and recent role in the dynamics and horizontal transfer of toxins. We searched for putative transposase genes by using TnpPred (29). We defined gene locations as toxin associated on the basis of the coding sequences in the five upstream and five downstream positions of genes encoding Cry, Vip, or Cyt proteins. Among the 2,252 genes in toxin-associated locations, 360 (15%) encoded transposases. This percentage is much higher than that of genes in all other locations on the genome (i.e., in loci not encoding toxins), 3% of which are associated with transposases (17,584/611,107). Transposases in the IS*4*, IS*6*, IS66, IS*605*, and Tn*3* families were particularly associated with toxin genes (Fig. 4).

### Multiple toxins from the same isolates have similar targets in the whole *B. thuringiensis* species*.*

**FIG 5:**
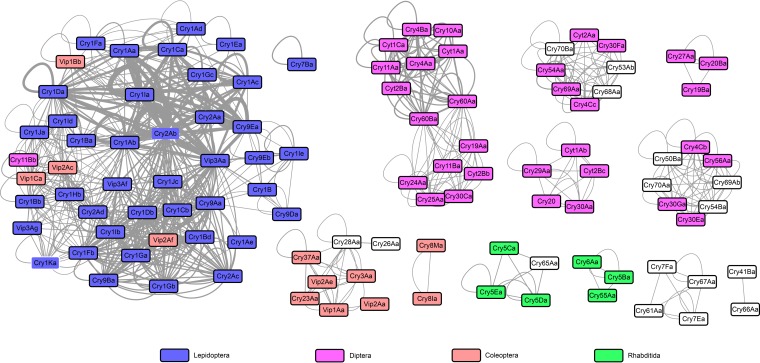
Co-occurrence network of all of the Cry, Cyt, and Vip proteins predicted in this study. Nodes represent different Cry proteins, and node colors represent their targets. Cry proteins that coexist in the same strain are represented by edge, and those that coexist more frequently are connected by thicker lines.

As described above, different toxin genes were frequently contained by the same *B. thuringiensis* strain. We studied how different toxins coexist in the same strain by constructing a co-occurrence network. Toxin target information was obtained from the literature (27, 30) and the *B. thuringiensis* toxin specificity database (http://www.glfc.cfs.nrcan.gc.ca/bacillus). In the network, nodes represent Cry, the background color of the node refers to the major target reported for Cry, and edges represent co-occurrence. The edge widths were weighted by the frequency of co-occurrence; some *cry* genes have multiple copies in the same strain, so many Cry proteins were self-connected. In the four most frequently reported host classes, almost all of the Cry proteins with similar targets were clustered in the same subnetwork, with very few exceptions (Fig. 5).

The largest subnetwork contained toxins in the Cry1, Cry2, Cry9, and Vip3 families, and all of these toxins were reported to have high toxicity for insects of the order Lepidoptera. Exceptions (with different host targets) in this subnetwork included Vip1 and Vip2, which are toxic to coleopteran insects, and Cry11Bb, which can kill dipteran insects. The other subnetwork containing Cry proteins toxic to lepidopteran insects was composed of only Cry7Ba. Toxins Cry3, Cry23, Cry37, Vip1, and Vip2 were also clustered into one subnetwork; all of these toxins were toxic to coleopteran insects. In contrast, Cry proteins killing dipteran insects were clustered into six different subnetworks. Cry4 and Cyt2 proteins were found in three of them, respectively, in agreement with experimental work that showed that combinations of these toxins play a crucial role in the targeting of dipteran insects (31). Two small subnetworks had toxins targeting nematodes that contain Cry5, Cry6, and Cry55. Physical linkage can explain some co-occurrence of toxin genes within a network, and we found that all of the toxins encoded by the same PAI have similar targets. Toxins encoded by BtPAI-I, BtPAI-II, and BtPAI-III, which include different members of the Cry1 and Cry2 families and Vip3, are toxic to Lepidoptera and co-occur in the same network. Toxins of the Cry4, Cry10, Cry11, and Cyt families encoded by BtPAI-IV and BtPAI-V can kill Diptera and were also clustered in the same subnetwork.

### Plasmids with the *orf156*/*orf157* minireplicon plays a crucial role in the formation and evolution of *B. thuringiensis* in clade 2.

**FIG 6:**
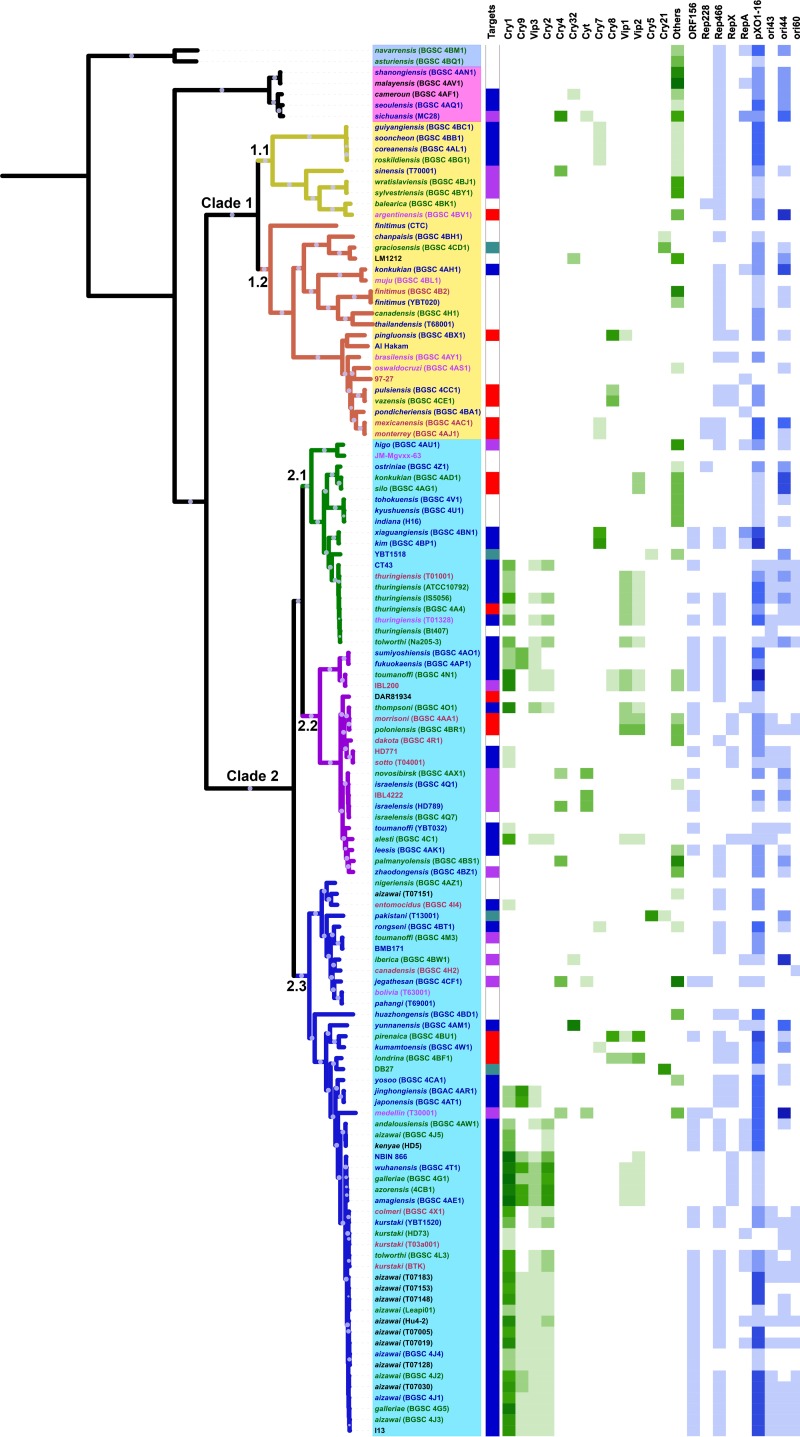
Distribution of *cry* genes and plasmids in *B. thuringiensis*. The tree shown is similar to that in Fig. 1. The innermost line represents target hosts of *B. thuringiensis* strains, blue for Lepidoptera, purple for Diptera, dark green for nematodes, and red for Coleoptera. In the heat map on the right, shades of green represent the numbers of genes for insecticidal proteins and shades of blue represent the numbers of Rep proteins encoded by genes on plasmids.

Previous genomic analysis of *B. thuringiensis* showed that most toxin genes were on megaplasmids with sizes of >100 kbp (32, 33). We explored the distribution of megaplasmids among all *B. thuringiensis* strains by studying the minireplicons they contained as described previously (32) and tried to find out the relationship between plasmid distributions and loss and gain of *B. thuringiensis* toxins (Fig. 6). Of the six types of minireplicons, three (*ori44*, *pXO1-14*/*pXO1-16*, and *rep466*) had very wide distributions within and between clades of the *B. cereus* group (Fig. 6; Fig. S1). Moreover, the *orf156*/*orf157* minireplicon, which was first reported to be essential for replication of the large plasmid pBtoxis (34), was contained only by *B. thuringiensis* in clade 2 (Fig. S1). In lineage 2.1, strains clustered close to serovar thuringiensis contained *orf156*/*orf157* plasmids. In lineage 2.2, strains with this plasmid showed a dispersed distribution on the phylogenetic tree, while in lineage 2.3, strains on the branch containing serovars kurstaki and aizawai had this plasmid (Fig. 6).

We studied the association between the toxin genes and the *orf156*/*orf157* minireplicon by analyzing all of the available *B. thuringiensis* strains with complete genome sequences. BtPAI-I is contained by plasmids with *orf156*/*orf157*, such as pCT281, pIS56-285, pBMB299, and pBMB300 from *B. thuringiensis* strains CT-43, IS5056, BTK, and YBT-1520, respectively. In HD-789, BtPAI-IV and BtPAI-V and another two toxin genes were all on plasmid pBTHD789-3, which has the *orf156*/*orf157* minireplicon. We also studied the synteny of these plasmids with complete genomes and other putative plasmids with similar minireplicons from their draft assemblies. We found that plasmids harboring similar PAIs usually have similar structures. Compared to pCT281, almost all of the plasmids with BtPAI-I or BtPAI-II have similar structures (Fig. S4A). The situation is the same for plasmids having BtPAI-IV and BtPAI-V in *B. thuringiensis* of lineage 2.2, such as serovars israelensis and novosibirsk (Fig. S4B).

10.1128/mBio.00822-17.4 FIG S4  Synteny analysis of plasmids containing the *orf156*/*orf157* minireplicon based on complete and draft genomes. (A) Plasmids having BtPAI-I or BtPAI-II. From the innermost circle to the outermost circle, pCT281; pIS56-285 (thuringiensis); pBMB293 (kurstaki); pBMB29 (kurstaki); and plasmids from different strains of sumiyoshiensis, fukuokaensis, andalousiensis, galleriae, galleriae, tolworthi, tolworthi, tolworthi, toumanoffi, thompsoni, colmeri, thuringiensis, aizawai, aizawai, aizawai, aizawai, aizawai, NBIN-866, and BtPAI-I/II are represented (2). Plasmids of *B. thuringiensis* are toxic to *Diptera*. From the innermost circle to the outermost circle, pBtoxis (israelensis); pBTHD789-3 (israelensis); and plasmids from novosibirsk, medellin, and IBL4222 (israelensis-like) are represented. Download FIG S4, PDF file, 0.5 MB. Copyright © 2017 Zheng et al. 2017 Zheng et al. This content is distributed under the terms of the Creative Commons Attribution 4.0 International license.

We combined the information about *B. thuringiensis* strain targets, major toxin genes, and megaplasmids to investigate the evolution and dynamics of pathogenicity of *B. thuringiensis* (Fig. 6). In clade 1, the only toxin gene with activity on Lepidoptera was *cry7Ba*. In clade 2, strains targeting Lepidoptera insects were mainly in lineages 2.1 and 2.3, which mostly contained the Cry1, Cry2, and Vip3 toxins and had *orf156*/*orf157* plasmids. Diptera-toxic strains were distributed discretely around the phylogenetic tree and contained mainly Cry4 and Cyt proteins, with some strains carrying Cry32. Among them, strains in clade 2 had *orf156*/*orf157*-like plasmids. In strains targeting coleopteran insects, those in clade 1 usually had genes encoding Cry8 and those in clade 2 additionally had genes for Vip1 and Vip2. Only four strains were inferred to be toxic to nematodes, and they were not clustered together on the phylogenetic tree. The specific toxin genes that they contained included *cry5*, *cry6*, *cry21*, and *cry55*. We infer that plasmids with *orf156*/*orf157* minireplicons are the most important vectors of the HGT contributing to the formation and dynamics of pathogenicity of *B. thuringiensis* in clade 2, especially those toxic to Lepidoptera and Diptera insects.

## DISCUSSION

As reported previously, *B. thuringiensis* was found in all clades of the *B. cereus* group and cannot be separated from other members of this group by phylogenetic analysis; in other words, it does not form a monophyletic group (24, 35) (Fig. S1). Since this species is defined by the expression of *cry* genes, a single event, namely, loss of the plasmid carrying *cry* genes, can result in a change in species designation. A substantial number of strains designated *B. thuringiensis* in this study carried no *cry* genes with a match to a protein in the database; subsequent examination by optical microscopy showed that they also lacked crystal inclusions, indicating that they are, in fact, *B. cereus* (Fig. S5). Misidentification and contamination have inflated the reporting of *B. thuringiensis* strains in unusual clades (16). A previous study has shown that “*B. thuringiensis”* serovars bolivia, vazensis, and navarrensis meet the description of *B. weihenstephanensis* (clade 3), although expression of crystal inclusions was not investigated (36); serovar navarrensis, from the evidence of this study, is not *B. thuringiensis* (Fig. 6). Although this study found fewer strains with no toxin genes predicted in clade 2 than in other clades, this is, in part, an artifact of the study design, which focused on strains described as *B. thuringiensis*. In previous studies, *B. cereus* or Cry null strains were widely distributed in all of the major clades (24), although *B. thuringiensis* was more common in clade 2, especially within some lineages (16).

10.1128/mBio.00822-17.5 FIG S5  Optical microscopy of *B. thuringiensis* strains with no toxin genes predicted from their genome sequences. Download FIG S5, PDF file, 0.4 MB. Copyright © 2017 Zheng et al. 2017 Zheng et al. This content is distributed under the terms of the Creative Commons Attribution 4.0 International license.

Phylogenetic analysis and ANI results showed that members of clade 2 represent a good bacterial species, in comparison to the other clades (Fig. 1B and 2). Clade 2 is quite genetically coherent, with an ANI among its strains of >95%; i.e., within the 95 to 96% boundary suggested in reference 37 as a new standard for defining bacterial species. Clade 2 contains many strains with high levels of toxicity to insect pests and which have been used as pesticides, including those from serovars thuringiensis, kurstaki, galleriae, aizawai, tenebrionis, and israelensis (38). The facts that clade 2 contains the strains that are the most effective invertebrate pathogens and contains three lineages dominated by Cry toxin producers (16) indicate that the typical *B. thuringiensis* strains with high levels of toxicity to insects originated from the same ancestor and evolved into three sublineages, respectively. On the basis of phylogenetic analysis, three subspecies could be proposed, i.e., lineages 2.1, 2.2, and 2.3.

It follows that Cry-producing strains in clades 1 and 3 may not warrant the designation “Thuringiensis”; their genetic background is distinct from invertebrate specialists (Fig. 1), and they appear to be ecologically distinct. Strains in clade 3 are closely related to *B. weihenstephanensis*, a species that is likely to be primarily saprophytic on the basis of the fact that it is psychrotolerant, is almost exclusively isolated from soil or plant material, and contains relatively few genes encoding VFs (21, 39, 40). In contrast, the vast majority of serious vertebrate infections are caused by isolates in clade 1 or the “Anthracis” clade, and members of this clade have the greatest potential to cause food poisoning in humans (16, 40). It seems likely that any invertebrate-specific toxins carried by strains in clades 1 and 3 have been acquired by recent HGT events; strains in these clades contain relatively few toxins at loci that are relatively unusual, i.e., not associated with *orf156*/*orf157*-containing megaplasmids. The fact that a large number of isolates originally designated thuringiensis in clades 1 and 3 appear to contain no *cry* genes at all also suggests that carriage of mobile elements with these genes might be relatively unstable, although we cannot exclude the role of contamination or misclassification here.

The co-occurrence of multiple toxins with similar invertebrate targets was typical for most *B. thuringiensis* strains and was particularly common in clade 2. Co-occurring toxins could either have similar protein structures or be derived from very different toxin classes. For instance, many strains in clade 2 with toxicity to Lepidoptera contained Cry1 toxins with a protein sequence homology of >78%, which is the boundary representing the second rank in the nomenclature of Cry toxins (41). Toxins in the same class may have similar receptors and modes of action. Nevertheless, accumulation of similar toxins in the same isolate can improve the speed with which some hosts are killed or expand the insecticidal spectrum among some phylogenetically related insects (42), as has been the practice with currently genetically engineered crops targeting lepidopteran pest complexes (43). In other cases, co-occurring toxins targeting Lepidoptera can be derived from a range of classes, such as Cry2 and Cry9 (with identities to those of Cry1 of <45%) or Vip3, which has no similarity to any Cry proteins. These toxins have different modes of action (44). Strains with putative toxicity to Diptera contained toxins of the Cry4, Cry10, Cry60, and Cyt families, and the identities of their protein sequences are <45%. Strains expressing coleopteran-active toxins usually contained the binary toxins Vip1/Vip2 and some Cry toxins, such as Cry3, Cry23, and Cry37. Combining toxins with different modes of action can lead to synergistic interactions, i.e., increased killing power per unit of protein, and so can provide an immediate selective benefit (45–48). Different toxins with different modes of action on similar insects can reduce the rate of evolution of resistance in targets, while Cyt toxins have the ability to mask genotypic resistance to other toxins (31, 49, 50); thus, combinations of toxins may provide a benefit in the long term or have evolved in response to resistance in some hosts (51).

Thus, synergism, extended host range, or improved resilience to resistance may have contributed to the co-occurrence of multiple toxins with broadly similar phylogenetic targets in single strains. This adds weight to the argument that *B. thuringiensis* strains, especially those in clade 2, represent specialized pathogens. This co-occurrence implies a continuous evolutionary history in hosts of a particular order. Thus, many strains must have undergone multiple successful cycles of infection in a restricted range of hosts. Some strains, such as *B. thuringiensis* serovar kurstaki ST8, may have particular adaptations (the ability to colonize plants) that enable them to infect a subset of hosts with greater efficiency (21), in addition to the Cry toxins described here.

Some *B. thuringiensis* toxins have been reported to have cross-order activity (30). For example, Cry2 toxins have been reported to be highly toxic to both Lepidoptera and Diptera insects. In our network analysis, all of the members of this family were in the subnetwork active against Lepidoptera. We suggest Lepidoptera insects as the original targets of Cry2 and Diptera insects as extended targets. Actually, each toxin with cross-order activity was found in a single subnetwork, which suggests that the alternative targets may have been formed by recent host range extension.

In addition, toxin network analysis may provide a powerful tool for predicting the virulence of newly described toxins with no known host affiliation. For example, in several subnetworks, some Cry proteins, such as Cry29, Cry50, Cry53, Cry68, and Cry70, had no reported target. We hypothesize that these toxins may be toxic to dipteran insects since they are contained within dipteran subnetworks. Actually, Cry29A was reported to synergize the toxicity of Cry11Bb against *Aedes aegypti* (Diptera: Culicidae), and Cry50Aa, Cry50Ba, Cry54Ba, Cry68Aa, and Cry70Aa were all found in some strains with high toxicity against mosquitoes (52–55). Similarly, in the coleopteran subnetwork, Cry26 and Cry28 had no reported toxicity; we suspect that they may target coleopteran hosts. Two small subnetworks had toxins targeting nematodes; again, the toxin Cry65Aa is likely to be active against this group of hosts, as it belongs to the same network as proteins such as Cry5 with well-described nematode toxicity (56).

Strains with complete genome sequences showed multiple toxins located in different positions on the same plasmid or different plasmids. Ten toxin genes, including seven *cry* and three *cyt* genes on three PAIs, were distributed separately on the same plasmid of HD-789 (57). In CT-43 and HD-1, five toxins were encoded by genes from two different plasmids, with four of them on the larger one and the other on a smaller one, respectively (58). In MC28, 11 toxin genes were contained by three different plasmids (53), and in IS5056, nine toxins were encoded by genes on four different plasmids (59). The range of loci encoding toxins implies that multiple toxins in the same genome were gained by multiple independent HGT events.

Plasmids play crucial roles in conferring pathogenicity on many pathogens (60). In the *B. cereus* group, genes encoding anthrax toxins of *B. anthracis* and those encoding emetic toxin of some *B. cereus* were also reported to be on plasmids (61, 62). All of the plasmids carrying these toxin genes have two minireplicons, *pXO1-16/pXO1-14* and *repX*, which belongs to one type of the *tubZ/tubR* class (32). The former class of minireplicon was carried by most of the strains of the *B. cereus* group but not by bacteria outside this group (Fig. S1). They are commonly combined with other types of minireplicons to enable the replication of megaplasmids (32, 63). Many megaplasmids carrying *B. thuringiensis* toxin genes also contained this kind of minireplicon; moreover, they harbored *orf156*/*orf157* belonging to another *tubZ/tubR* minireplicon. As reported previously, megaplasmids in the *B. cereus* group were formed by integrating smaller plasmids (32). We infer that the ancestral plasmids containing *pXO1-16/pXO1-14* were important for the formation of the *B. cereus* group, as they integrated with different plasmids to make the host strains with different toxicities. For example, the clade 2 *B. thuringiensis* strains toxic to Lepidoptera were formed by the integration of one plasmid with *orf156*/*orf157* and another plasmid with *pXO1-16/pXO1-14*.

The distribution of key virulence genes indicates that repeated interactions with a subset of hosts (e.g., insect orders) led to the accumulation of multiple toxins by successful clones and by successful PAIs. This accumulation may be one of the strategies that *B. thuringiensis* has used to combat host resistance or to increase its pathogenicity. While *B. thuringiensis* is not a monophyletic group, the vast majority of isolates were concentrated into a single clade and all of the strains in this clade contained multiple invertebrate VFs, suggesting that a phylogeny-based revision of the nomenclature of the *B. cereus* group could be valuable. Plasmids play a crucial role in HGT, and notably, plasmids carrying PAIs with multiple invertebrate toxins were restricted to this one clade. If clade 2 contains invertebrate specialists that are defined by their plasmid complement and by their genome, this leads to the question of what the evolutionary and ecological relationships between closely related *B. thuringiensis* and *B. cereus* strains within clade 2 are. Potentially, *B. cereus* clones in clade 2 represent specialist lineages that exploit invertebrate/vertebrate cadavers. Alternatively, *B. cereus* may persist as a toxin cheater and depend on coinfection with Cry-producing *B. thuringiensis* to access host resources (64, 65).

## MATERIALS AND METHODS

### Bacterial strains.

To investigate the genomics of *B. thuringiensis*, antiserum standard strains of each serovar and some additional strains of serotypes H5ab, H5ac, and H7 were obtained from the *Bacillus* Genetic Stock Center or the Institute Pasteur. Other strains used in this study were isolated and kept in our laboratory (Table S1). Genomic DNA for sequencing was prepared as described previously (33).

### Genome sequencing and assembly.

Samples were sequenced by using multiplexed libraries with Illumina HiSeq 2000, 2500, or 4000 to produce pair-end reads with lengths of 100, 125, and 150 bp. For each sample, reads were assessed with the FastQC tool (http://www.bioinformatics.babraham.ac.uk/projects/fastqc/) and low-quality reads were filtered by Quake (66). All of the assemblies were performed by ABySS with different k-mers (67); the best assembly for each strain, with the largest scaffold N50, was annotated by Prokka (68).

### Phylogenetic analysis.

All *B. thuringiensis* protein sequences were clustered by the MCL algorithm with an inflation value of 2, after an all-against-all BlastP search with an E value of <10^−5^ (69). Coding sequences of the single-copy core protein in each cluster were aligned by MUSCLE (70), and the alignment was trimmed by TrimAl (71). All alignments were concatenated with an in-house Perl script. The ML phylogenetic tree was constructed with RAxML by using the generalized time reversible (GTR) model and a gamma distribution to model site-specific rate variation (72). Bootstrap support values were calculated from 500 replicates.

For the whole *B. cereus* group, all of the genome sequences available were obtained from GenBank. Assemblies with a scaffold N50 of <50 kbp were filtered out. The SNPs of the core sequences of all of the genomes, including those sequenced in this study and those downloaded from GenBank, were obtained by the northern Arizona SNP pipeline (https://github.com/TGenNorth/NASP). An ML phylogeny was constructed by FastTree with a GTR+CAT (GTR with per-site rate categories) model of approximation for site rate variation (73). Bootstrap support values were calculated from 500 replicates.

*B. thuringiensis* population structure was defined with the hierBAPS module of the BAPS software, which delineates the population structure by nested clustering (74). Three independent iterations with upper population sizes of 8, 16, and 24 were used to obtain optimal clustering of the population.

### Analysis of toxins and plasmids.

Genes for Cry, Vip, and Cyt proteins were predicted from all of the *B. thuringiensis* genome sequences used in this study by the second version of BtToxin_scanner (75). It predicted all of the putative toxins on the basis of the homologies to all of the reported toxins and classified them on the basis of their sequence identities to those from the public database by using the nomenclature described by Crickmore et al. (41). Toxin target information was collected from the *B. thuringiensis* toxin specificity database (http://www.glfc.cfs.nrcan.gc.ca/bacillus) and from literature sources (27, 30). The frequency of co-occurrence of each pair of toxins was explored by using an in-house Perl script. The co-occurrence network was constructed and visualized by the Cytoscape software (76). Toxins of *B. thuringiensis* strains that were reported to contain two or more toxin genes were collected from the *B. thuringiensis* toxin database (http://www.btnomenclature.info). Co-occurrence analysis was performed as described above.

Minireplicons that contain the origin of replication and genes encoding replication proteins were used to analyze the distribution and dynamics of plasmids in the *B. cereus* group as described in our previous work. We focused on putative plasmids with similarity to those mentioned in our previous work (32).

### Availability of supporting data.

The data sets supporting the results of this article are included in the supplemental material.
