# Supplementary material for: Comparative Genomics of Bacillus thuringiensis Reveals a Path to Specialized Exploitation of Multiple Invertebrate Hosts
Source: mBio. 2017 Aug 8;8(4):e00822-17. doi: 10.1128/mBio.00822-17 (PMC5550751; doi:10.1128/mBio.00822-17)
Supplement: TABLE S2 [file mbo004173420st2.docx]

**Table S2** Detail about *cry* proteins predicted in *Bacillus thuringiensis*

| Stain | Serovar | Serotype | *cry* genes |
| --- | --- | --- | --- |
| **Clade 1.1** | | | |
| 4BC1 | *guiyangiensis* | 43 | Cry7Ba |
| 4BB1 | *sooncheon* | 41 | Cry7Ba |
| 4AL1 | *coreanensis* | 25 | Cry7Ba |
| 4BG1 | *roskildiensis* | 45 | Cry7Ba |
| 4BV1 | *argentinensis* | 58 | Cry73Aa |
| **Clade 1.2** | | | |
| YBT020 | *finitimus* | 2 | Cry26Aa, Cry28Aa |
| 4B2 | *finitimus* | 2 | Cry26Aa, Cry28Aa |
| 4CD1 | *graciosensis* | 66 | Cry21Ba-like(2) |
| 4BX1 | *pingluonsis* | 60 | Cry8Ab |
| 4AS1 | *oswaldocruzi* | 38 | Cry41Ba, Cry66Aa |
| 4CC1 | *pulsiensis* | 65 | Cry8Ia, Cry8Ma |
| 4CE1 | *vazensis* | 67 | Cry8Ma, Cry8Ia |
| 4AC1 | *mexicanensis* | 27 | Cry7Ab |
| 4AJ1 | *monterrey* | 28ab | Cry7Ab |
| **Clade 2.1** | | | |
| 4BN1 | *xiaguangiensis* | 51 | Cry7Ba |
| 4BP1 | *kim* | 52 | Cry7Ba |
| YBT1518 |  |  | Cry55Aa, Cry5Ba, Cry6Aa |
| CT43 |  |  | Vip3Aa, Cry2Ab, Cry1Aa, Cry1Ia, Cry2Aa, Cry1Ba |
| T01001 | *thuringiensis* | 1 | Cry1Ab, Cry1Ba, Vip1Bb, Vip2Aa |
| T01328 | *thuringiensis* | 1 | Vip3Aa, Cry2Ab, Cry1Aa, Cry1Ia, Cry2Aa, Cry1Ba, Vip1Bb, Vip2Aa |
| 4A3 | *thuringiensis* | 1 | Cry60Ba, Cry60Aa |
| 4A4 | *thuringiensis* | 1 | Cry1Ba, Vip1Bb |
| IS5056 | *thuringiensis* | 1 | Vip3Aa, Cry2Ab, Cry1Aa, Cry1Ia, Cry2Aa, Cry1Ab, Cry1Ba, Vip1Bb, Vip2Aa |
| ATCC10792 | *thuringiensis* | 1 | Cry1Ab, Cry1Ba, Vip1Bb, Vip2Aa |
| Na205-3 | *tolworthi* | 9 | Vip3Aa, Cry2Ab, Cry1Aa, Cry1Ia, Cry2Aa, Cry1Ab, Cry1Ba, Vip1Ba, Vip2Aa |
| 4AU1 | *higo* | 44 | Cry19Ba, Cry20Ba, Cry27Aa |
| **Clade 2.2** | | | |
| 4AO1 | *sumiyoshiensis* | 3ad | Cry1Ie, Cry9Aa, Cry9Da, Vip3Aa |
| 4AP1 | *fukuokaensis* | 3ade | Cry1Ie, Cry9Aa, Cry9Da, Vip3Aa |
| 4N1 | *toumanoffi* | 11ab | Cry1Hb, Cry1Bb, Cry1Ab, Cry1Nc, Cry1Ja, Cry1Id, Cry1Da, Cry11Bb, Cry2Ad, Vip1Ca, Vip2Ac, Vip3Af |
| IBL200 |  |  | Cry1Hb, Cry1Bb, Cry1Ab, Cry1Nc, Cry1Ja, Cry1Id, Cry1Da, Cry2Ad, Cry8Ba, Vip1Ba, Vip2Ac, Vip3A |
| 4O1 | *thompsoni* | 12 | Cry1Hb, Cry1Bb, Cry1Ab, Cry1Fb, Cry1Ib, Cry1Ka, Cry2Ad, Vip1Ca, Vip2Ac, Vip3Af, Vip3Ag |
| 4AA1 | *morrisoni* | 8ab | Cry3Aa, Cry23Aa |
| 4BR1 | *poloniensis* | 54 | Cry3Aa, Cry23Aa, Cry28Aa, Vip1Aa, Vip2Ae |
| HD771 |  |  | Cry1Aa |
| T04001 | *sotto* | 4 | Cry1Aa |
| 4AX1 | *novosibirsk* | 24ac | Cry60Ba, Cry60Aa, Cry4Ba, Cyt2Ba, Cry11Aa, Cyt1Aa, Cry10Aa, Cyt1Ca |
| 4Q1 | *israelensis* | 14 | Cry60Ba, Cry60Aa |
| IBL4222 |  |  | Cry60Ba, Cry60Aa, Cry4Ba, Cyt2Ba, Cry11Aa, Cyt1Aa, Cry10Aa, Cyt1Ca |
| HD789 | *israelensis* | 14 | Cry60Ba, Cry60Aa, Cry4Ba, Cyt2Ba, Cry11Aa, Cyt1Aa, Cry4Aa, Cry10Aa, Cry4Ba, Cyt1Ca |
| YBT032 | *darmstadiensis* | 10ab | Cry1Ea |
| 4C1 | *alesti* | 3ac | Cry1Ae, Cry1Gb, Cry2Ab, Vip2Af, Vip3Aa |
| 4BS1 | *palmanyolensis* | 55 | Cry59Aa |
| 4BZ1 | *zhaodongensis* | 62 | Cry31Aa-like |
| **Clade 2.3** | | | |
| YBT1520 | *kurstaki* | 3abc | Vip3Aa, Cry2Ab, Cry1Aa, Cry1Ia, Cry2Aa, Cry1Ac |
| BTK | *kurstaki* | 3abc | Vip3Aa, Cry2Ab, Cry1Aa, Cry1Ia, Cry2Aa, Cry1Ab, Cry1Ac |
| HD73 | *kurstaki* | 3abc | Cry1Ac |
| T03a001 | *kurstaki* | 3abc | Cry1Ac |
| 4G5 | *galleriae* | 5ab | Vip3Aa, Cry2Ab, Cry1Aa, Cry1Ia, Cry1Ca, Cry1Da, Cry1Ac, Cry1Gc, Cry9Ea, |
| Leapi01 | *aizawai* | 7 | Vip3Aa, Cry2Ab, Cry1Aa, Cry1Ia, Cry1Ca, Cry1Da, Cry1Ia, Cry9Ea |
| Hu4-2 | *aizawai* | 7 | Cry1Ia, Cry9Ea, Vip3Ca |
| 4J1 | *aizawai* | 7 | Vip3Aa, Cry2Ab, Cry1Aa, Cry1Ia, Cry1Ca, Cry1Da, Cry9Ea |
| 4J2 | *aizawai* | 7 | Vip3Aa, Cry2Ab, Cry1Aa, Cry1Ia, Cry1Ca, Cry1Da, Cry9Ea, Cry9Ea |
| 4J3 | *aizawai* | 7 | Vip3Aa, Cry2Ab, Cry1Aa, Cry1Ia, Cry1Ca, Cry1Da, Cry1Ab, Cry9Ea |
| 4J4 | *aizawai* | 7 | Vip3Aa, Cry2Ab, Cry1Aa, Cry1Ia, Cry9Ea |
| 4J5 | *aizawai* | 7 | Cry1Ac, Cry1Ea, Cry1Ia, Cry2Aa |
| T07005 | *aizawai* | 7 | Vip3Aa, Cry2Ab, Cry1Aa, Cry1Ia, Cry1Ca, Cry1Da, Cry9Ea |
| T07019 | *aizawai* | 7 | Vip3Aa, Cry2Ab, Cry1Gc*, Cry1Ia, Cry1Ca, Cry1Da, Cry1Aa, Cry1Ac, Cry9Ea |
| T07030 | *aizawai* | 7 | Vip3Aa, Cry2Ab, Cry1Gc*, Cry1Ia, Cry1Ca, Cry1Da, Cry1Ab*, Cry9Ea, Cry9Ea* |
| T07128 | *aizawai* | 7 | Vip3Aa, Cry2Ab, Cry1Aa, Cry1Ia, Cry9Ea |
| T07148 | *aizawai* | 7 | Vip3Aa, Cry2Ab, Cry1Gc*, Cry1Ia, Cry1Ca, Cry1Da, Cry1Ab*, Cry9Ea |
| T07153 | *aizawai* | 7 | Vip3Aa, Cry2Ab, Cry1Aa, Cry1Ia, Cry1Ca, Cry1Da, Cry9Ea |
| T07183 | *aizawai* | 7 | Vip3Aa, Cry2Ab, Cry1Gc*, Cry1Ia, Cry1Ca, Cry1Da, Cry1Ab*, Cry9Ea |
| 4L3 | *tolworthi* | 9 | Vip3Aa, Cry2Ab, Cry1Gc*, Cry1Ia, Cry2Aa, Cry1Ac, Cry1Aa |
| 4X1 | *colmeri* | 21 | Vip3Aa, Cry2Ab, Cry1Gc*, Cry1Ia, Cry1Ac, Cry1Aa |
| 4AE1 | *amagiensis* | 29 | Cry1Db*, Cry1Ib, Cry9Aa*, Cry9Aa*, Cry9Aa, Vip3Af, Cry2Ab, Cry1Bd, Cry1Cb, Cry1Fb, Cry1Ga, Cry1Gb, Cry2Ab(1+2*), Cry2Ac, Vip1Aa, Vip2Af, Vip3Aa |
| 4CB1 | *azorensis* | 64 | Cry1Db*, Cry1Ib, Cry9Aa*, Cry9Aa*, Cry9Aa, Vip3Af, Cry2Ab, Cry1Bd, Cry1Cb, Cry1Fb, Cry1Ga, Cry1Gb, Cry2Ab(1+2*), Cry2Ac, Vip1Aa, Vip2Af, Vip3Aa |
| 4G1 | *galleriae* | 5ab | Cry9Aa*, Cry1Ib, Cry1Db, Cry1Fb, Cry1Ab(1+2*), Cry1Bd, Cry1Cb, Cry1Ga, Cry1Gb, Cry2Ab, Cry2Ac*, Cry9Ba*, Vip1Aa, Vip2Af, Vip3Aa, Vip3Af |
| 4T1 | *wuhanensis* |  | Cry1Bd, Cry1Cb, Cry1Db*, Cry1Fb, Cry1Ga, Cry1Gb, Cry1Ib, Cry2Ab(2+2*), Cry2Ac*, Cry9Aa, Cry9Ba(1+2*), Vip1Aa, Vip2Af, Vip3Aa, Vip3Af |
| HD5 | *kenyae* | 4ac | Cry1Ac, Cry1Ea, Cry1Ia, Cry2Aa |
| NBIN 866 |  |  | Cry1Ib, Cry1Db, Cry1Fb, Cry1Bd, Cry1Cb, Cry1Ga, Cry1Gb, Cry2Ac*, Cry2Ad*, Vip1Aa, Vip2Af, Vip3Af |
| 4AW1 | *andalousiensis* | 37 | Vip3Aa, Cry2Ab, Cry1Ia, Cry2Aa, Cry1Ac, Cry1Jc |
| I13 |  |  | Vip3Aa, Cry2Ab, Cry1Ia, Cry1Ca, Cry1Da, Cry1Ab, Cry1Gc, Cry9Ea, |
| T30001 | *medellin* | 30 | Cry29Aa, Cry30Aa, Cyt1Ab, Cyt2Bc |
| 4BT1 | *rongseni* | 56 | Cry7Ba |
| 4AT1 | *japonensis* | 23 | Cry1Ie, Cry9Da, Vip3Aa |
| 4AR1 | *jinghongiensis* | 42 | Cry1Ie, Cry9Da, Vip3Aa |
| 4W1 | *kumamtoensis* | 18ab | Cry7Ab |
| 4BU1 | *pirenaica* | 57 | Cry8Ba |
| 4AM1 | *yunnanensis* | 20ab | Cry32Aa |
| 4BD1 | *huazhongensis* | 40 | Cry8Ia, Cry8La |
| 4CF1 | *jegathesan* | 28ac | Cry60Ba, Cry60Aa, Cry11Ba, Cry19Aa, Cry24Aa, Cry25Aa, Cry30Ca, Cyt2Bb |
| 4I4 | *entomocidus* | 6 | Cry1Ba |
| 4BF1 | *londrina* | 10ac | Cry8Ka |
| T13001 | *pakistani* | 13 | Cry5Ca, Cry5Da, Cry5Ea, Cry65Aa |
| **Others** | | | |
| 4AF1 | *cameroun* | 32 | Cry32Aa-like |
| MC28 | *sichuansis* |  | Cry30Fa, Cry4Cc, Cry53Ab, Cry54Aa, Cry68Aa, Cry69Aa, Cry69Aa, Cry70Ba, Cyt2Aa |

Genes encoding toxins in the same background the shadow are in the same pathogenicity island.
