# Supplementary figures and images for: Comparative Genomics of Bacillus thuringiensis Reveals a Path to Specialized Exploitation of Multiple Invertebrate Hosts
Source: mBio. 2017 Aug 8;8(4):e00822-17. doi: 10.1128/mBio.00822-17 (PMC5550751; doi:10.1128/mBio.00822-17)

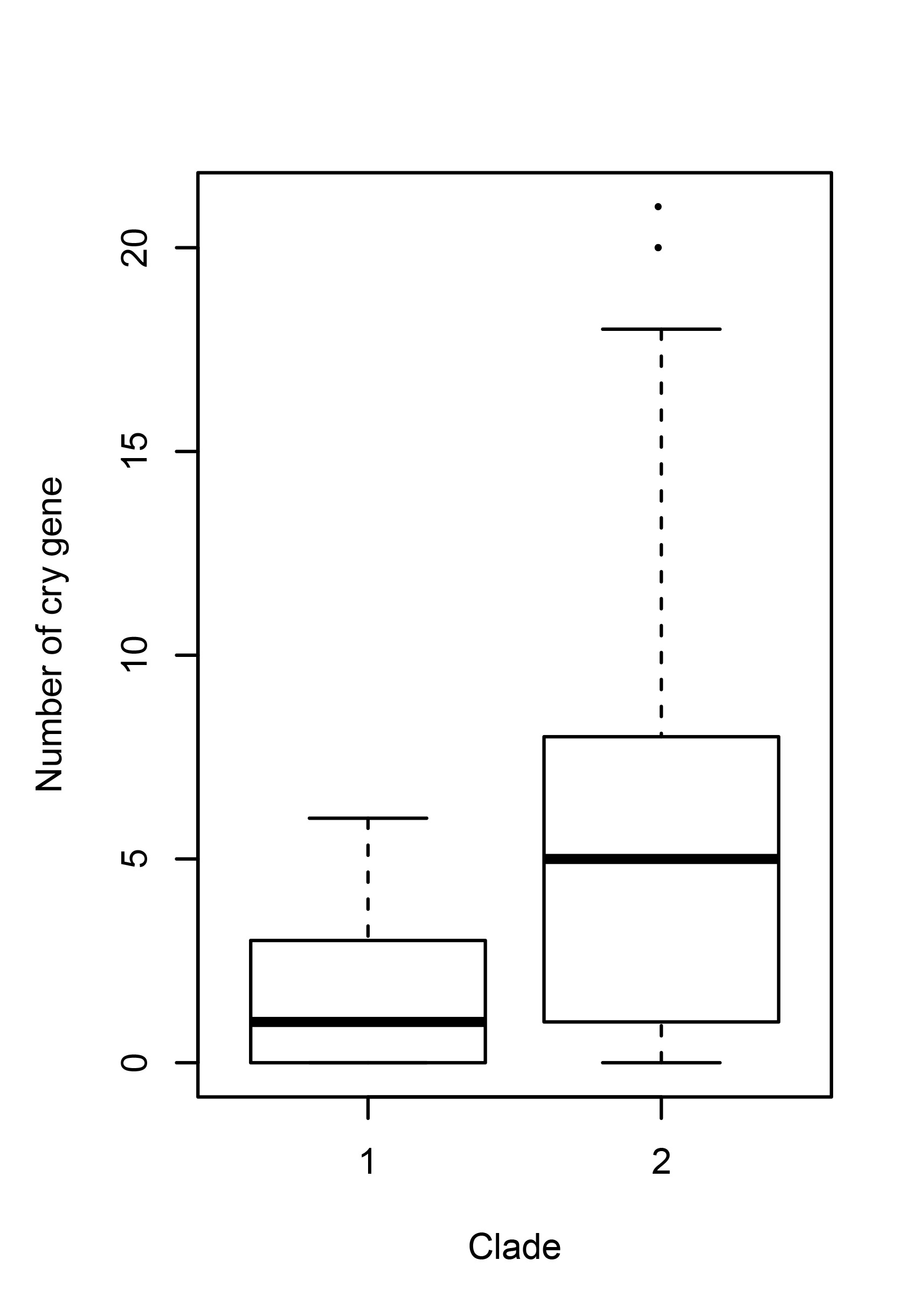

Supplement: FIG S2 [file mbo004173420sf2.tif]

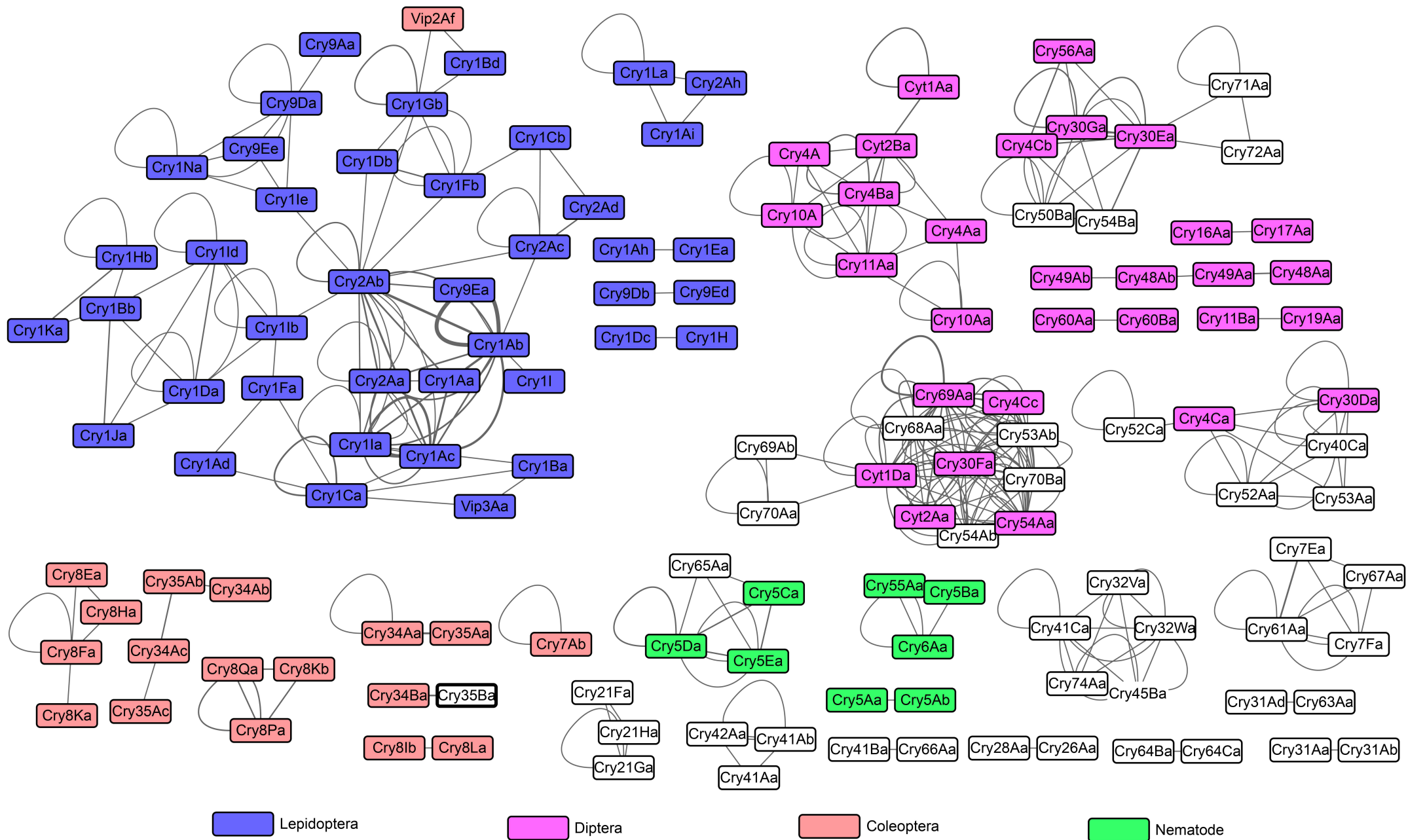

Supplement: FIG S3 [file mbo004173420sf3.pdf]

A

BtPAI-I/II

pCT281  
281,231 bp

B

pBTHD789-3  
224,872 bp

BtPAI-IV

BtPAI-V

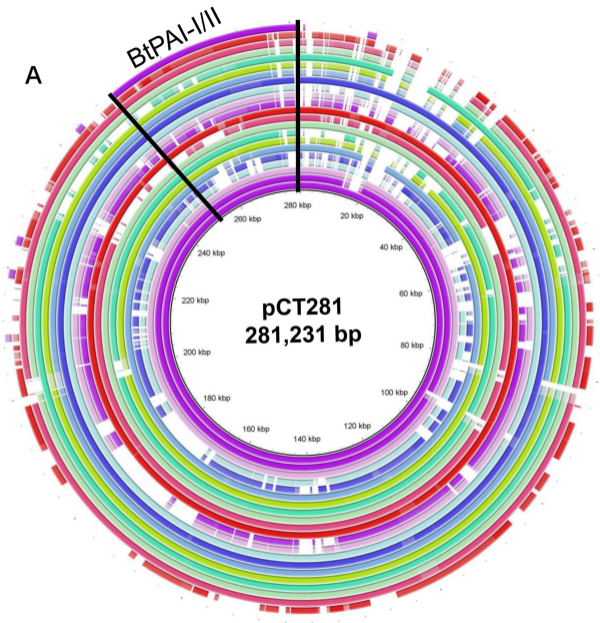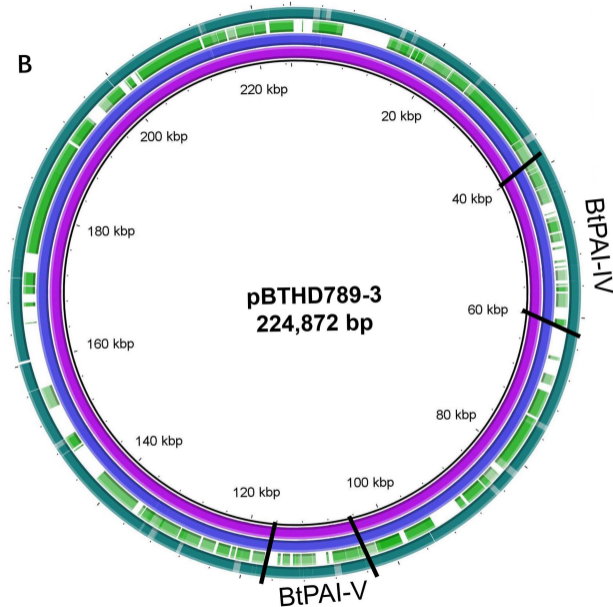

Supplement: FIG S4 [file mbo004173420sf4.pdf]

4BK1

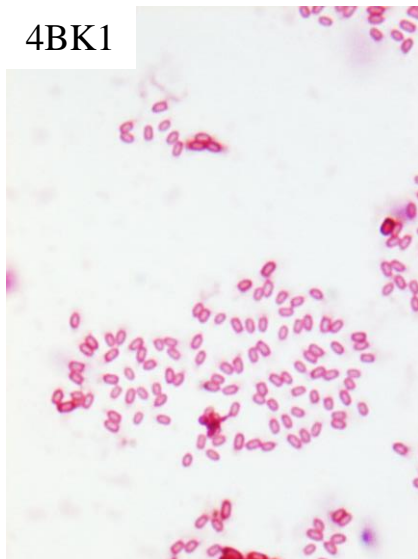

CTC

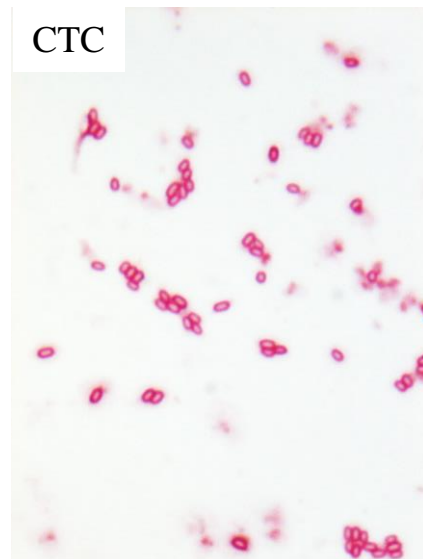

4BJ1

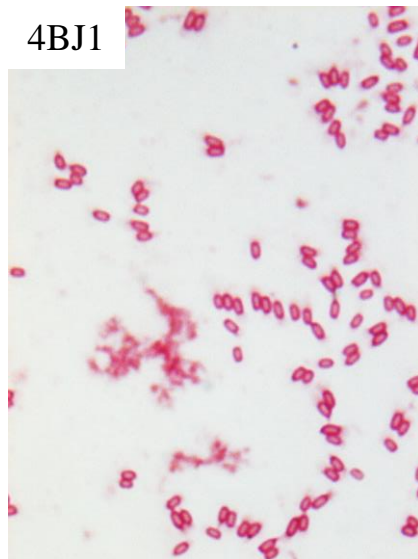

4BK1

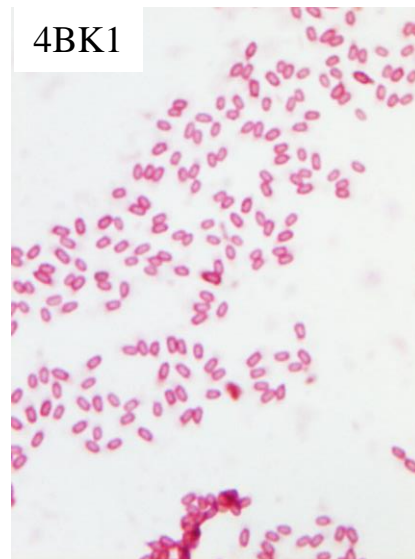

4H1

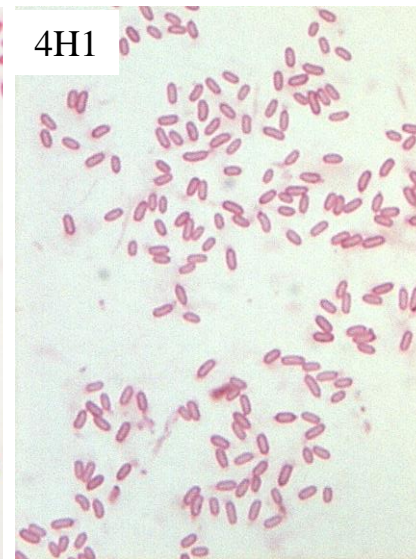

T68001

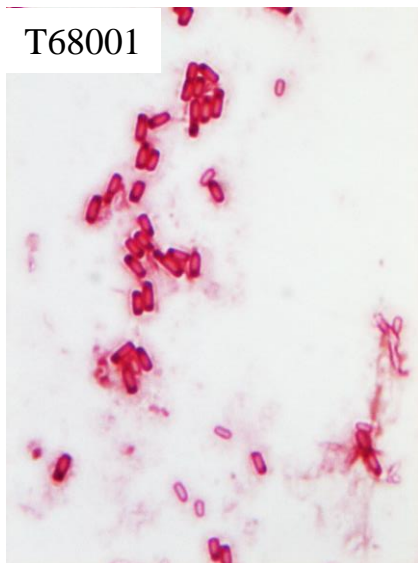

4AY1

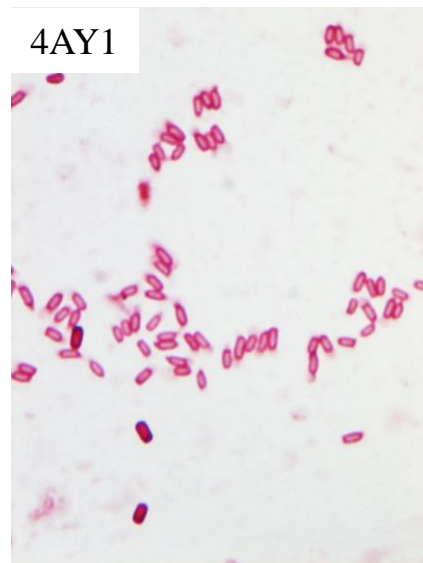

4AS1

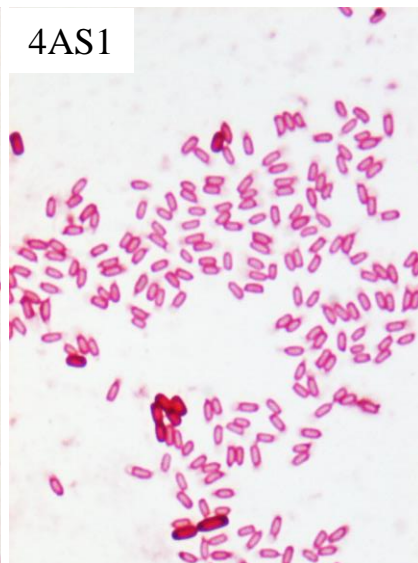

4BA1

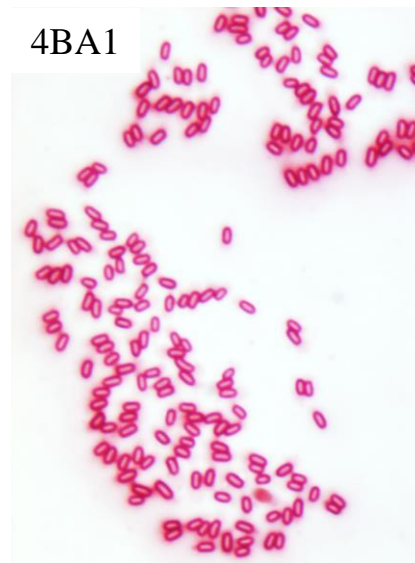

4BY1

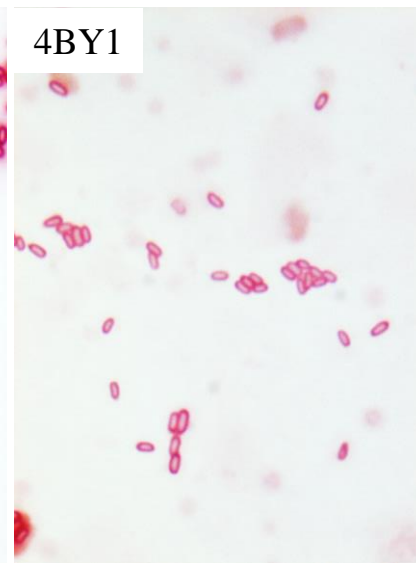

4Z1

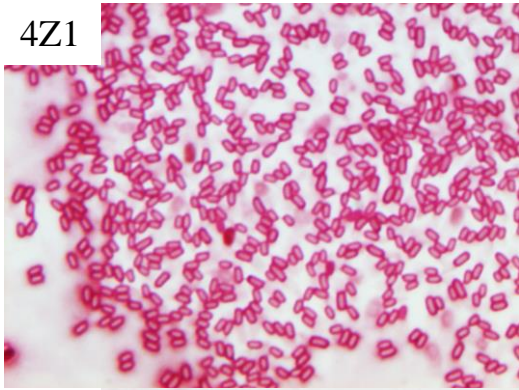

4V1

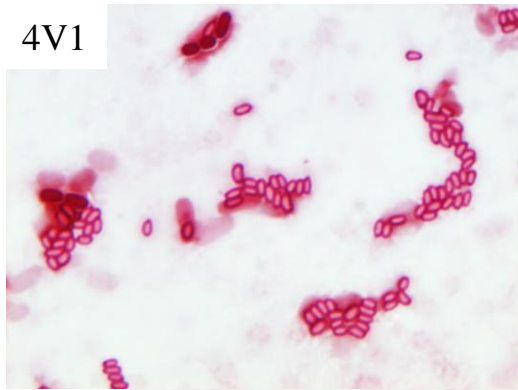

4U1

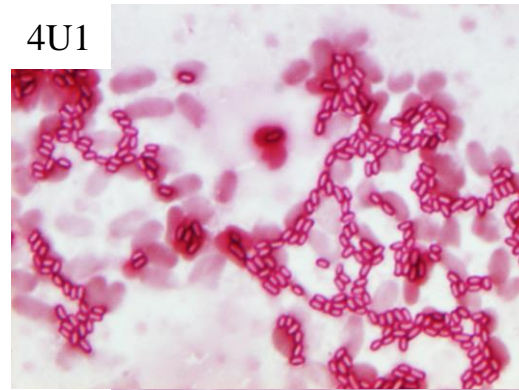

h16

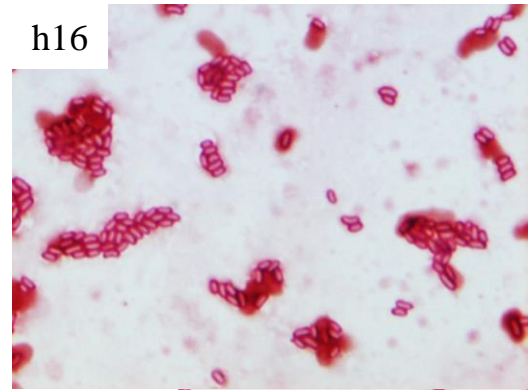

4R1

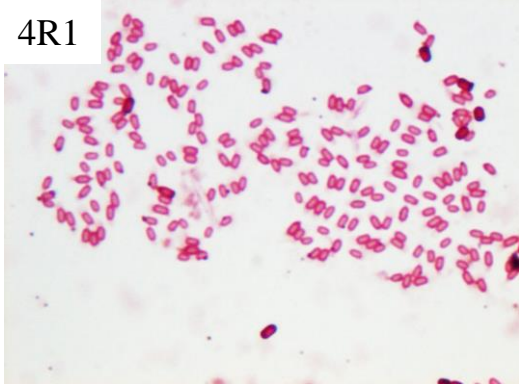

4AZ1

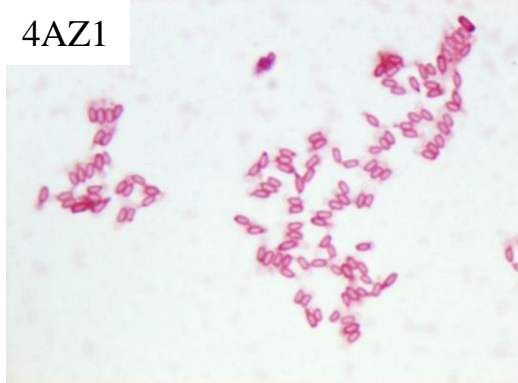

4H2

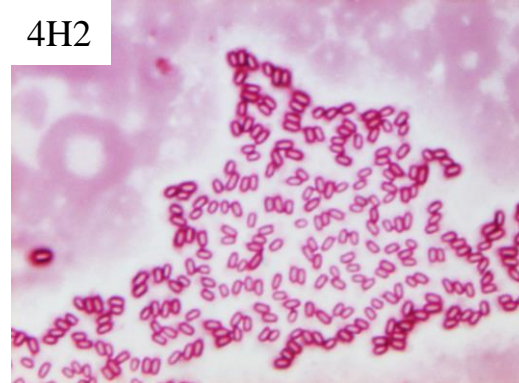

T69001

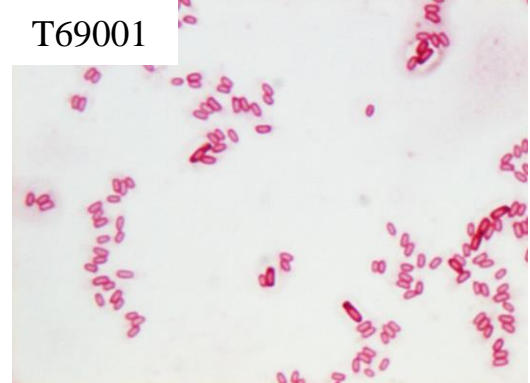

Supplement: FIG S5 [file mbo004173420sf5.pdf]
