# Supplementary material for: Comparative Genomics of Bacillus thuringiensis Reveals a Path to Specialized Exploitation of Multiple Invertebrate Hosts
Source: mBio. 2017 Aug 8;8(4):e00822-17. doi: 10.1128/mBio.00822-17 (PMC5550751; doi:10.1128/mBio.00822-17)
Supplement: FIG S1 [file mbo004173420sf1.pdf]

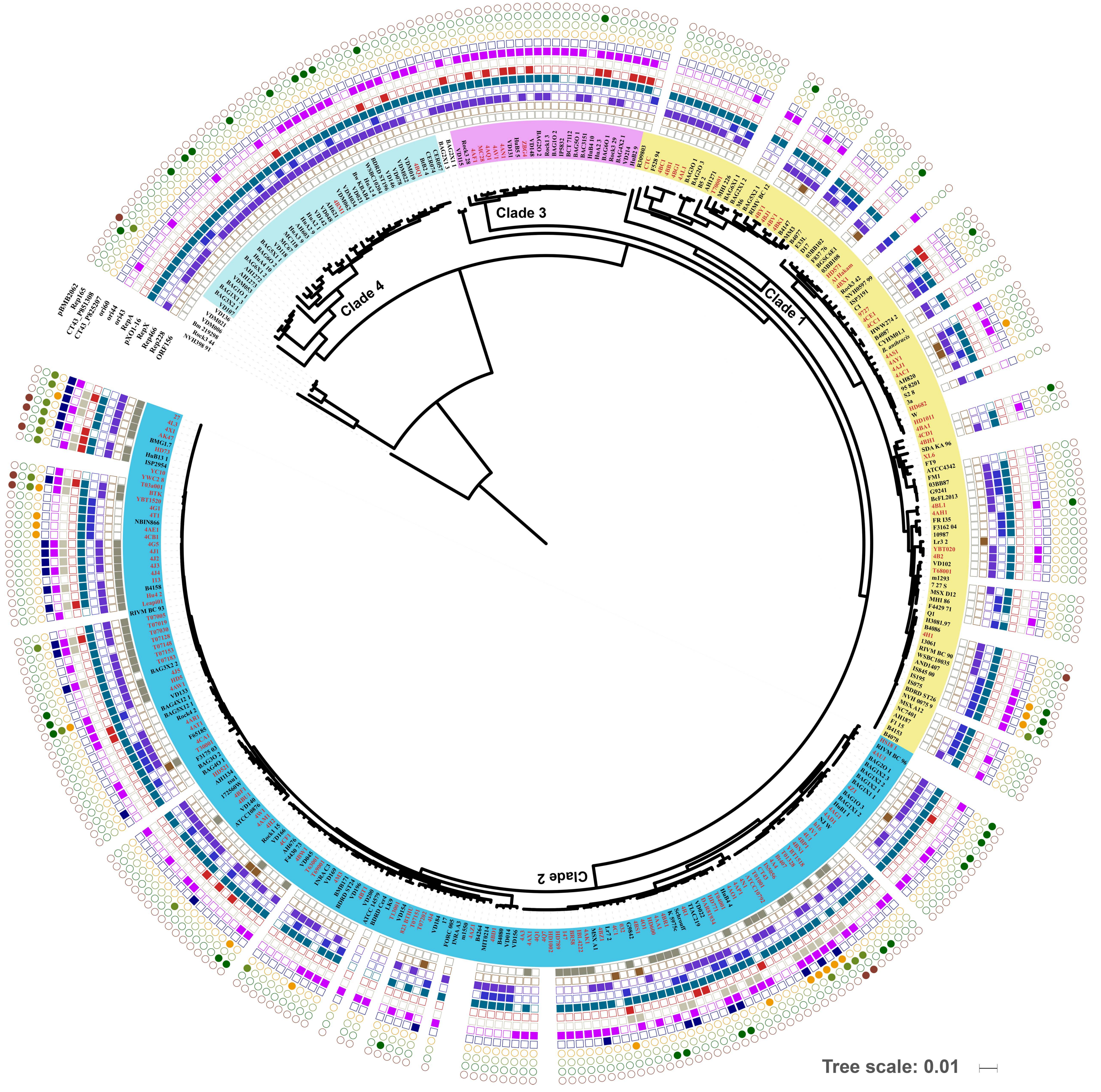
Clade 1
Clade 2
Clade 3
Clade 4
Tree scale: 0.01
